# Supplementary material for: Cost-Effectiveness of an Organized Lung Cancer Screening Program for Asbestos-Exposed Subjects
Source: Cancers (Basel). 2022 Aug 24;14(17):4089. doi: 10.3390/cancers14174089 (PMC9454930; doi:10.3390/cancers14174089)
Supplement: Supplementary file 1 [file cancers-14-04089-s001.zip › cancers-1846829-supplementary.pdf]

Supplementary Figure S1. Scatter plot of the main analysis: Population with occupational asbestos exposure, 1-year interval between low-dose thoracic computed-tomography scans, screening started at 55 years old and lifetime time horizon. ICER, incremental cost-effectiveness ratio; QALYs, quality-adjusted life years.

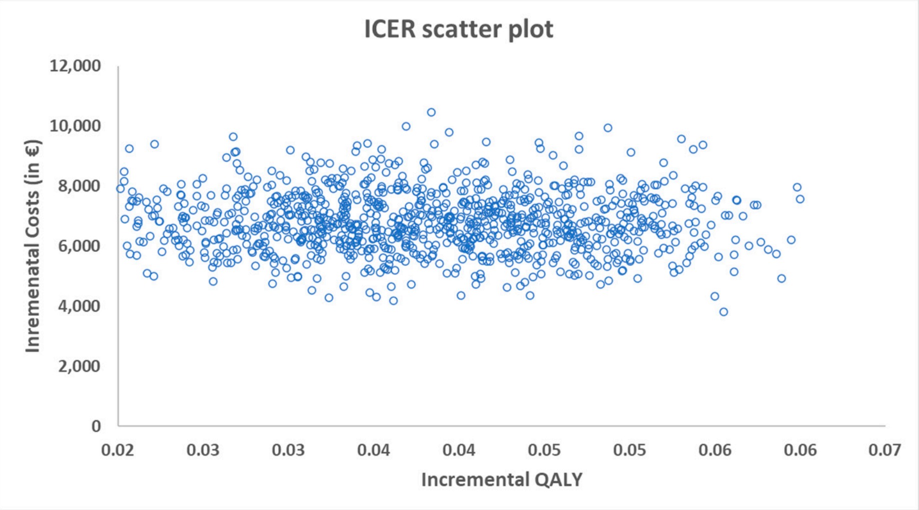

Supplementary Figure S2. Cost-effectiveness analysis curve

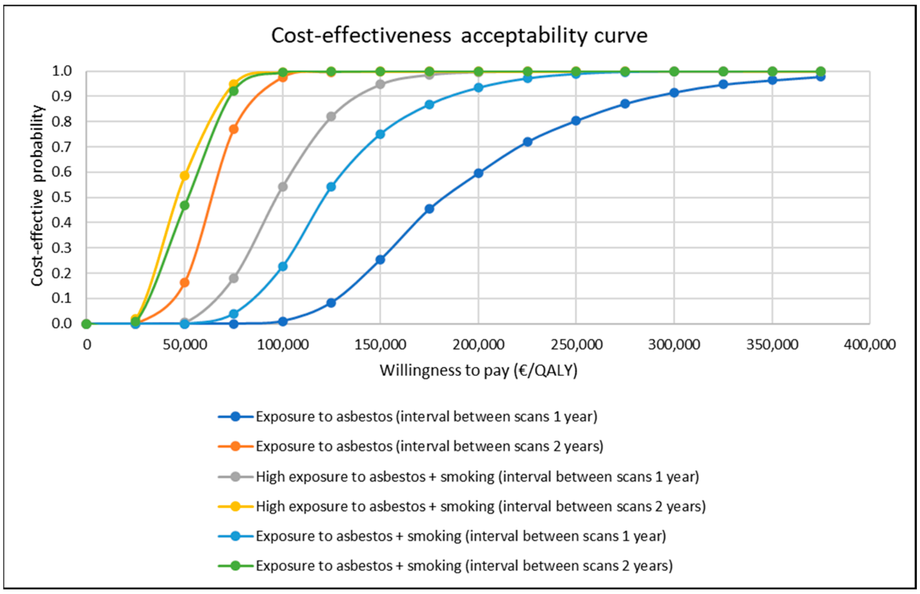

Supplementary Table S1. French General Population Mortality 2019 (INED).

French General Population Mortality 2019 (source : INED)	
No. of deaths per year per 1000 living persons	Age
2	45
2	46
2	47
2	48
2	49
3.2	50
3.2	51
3.2	52

3.2	53
3.2	54
4.9	55
4.9	56
4.9	57
4.9	58
4.9	59
7.5	60
7.5	61
7.5	62
7.5	63
7.5	64
10.6	65
10.6	66
10.6	67
10.6	68
10.6	69
18.1	70
18.1	71
18.1	72
18.1	73
18.1	74
18.1	75
18.1	76
18.1	77
18.1	78
18.1	79
57.3	80
57.3	81
57.3	82
57.3	83
57.3	84
57.3	85
57.3	86
57.3	87
57.3	88
57.3	89
183	90
183	91
183	92
183	93
183	94
183	95
183	96
183	97
183	98
183	99
183	100
183	101
183	102
183	103

183	104
183	105
183	106
183	107
183	108
183	109
183	110
183	111
183	112
183	113
183	114
183	115

Supplementary Table S2. Costs in Euros of ARDCO Cohort and Individual Non-Intervention-Subject-Initiated Care

Option	ARDCO cohort	Individual subject care
**Option 1** [1] **French National Health Insurance data main analysis (N = 10,560)**		
Total costs (from common classification of medical procedures)	75,886 €	7 €
Total costs (from homogeneous patient group, private facility hospitalizations, category 4)	189,798 €	19 €
Total costs (FNHI)		26 €
**Option 2**	167,606	157,823
**Questionnaire response data (sensitivity analysis) (N = 3,287)**	112,202	99,531
Total cost (Questionnaire)		170 €

[1] Care use was calculated using the extraction of the FNHI data for homogeneous patient group (GHMs, = private hospitalizations) and common classification of medical procedures (CCAM codes) for respiratory procedures performed in the private sector, during the year 2018.

Supplementary Table S3. Costs Generated by Lung Cancer Screening and Management of False-Positive Findings.

Detailed action billed		Cost
Screening for lung cancer		
Subject selection		46 €
Low-dose thoracic CT scan		86 €
Examination	CT scan without contrast injection	30 €
Surcharge Y (+15.8%)	Radiography by a radiologist, pneumologist or rheumatologist	
Image-archiving		0.78 €
Fixed-rate technical costs		30 – 62 €
Pneumology consultation		46 €
Organizational costs		11 €
**Total cost (mean per person)**		189 €
False-positive findings		
CT scan with contrast injection	100%	86 €
Brain CT scan	80%	86 €
Abdominal CT scan	80%	86 €
Bronchial fibroscopy	80%	96 €

Positron-emission tomography scan	80%	840 €
Pulmonary function tests	24.6%	137 €
Mediastinoscopy	2%	2221 €
Scan-guided puncture	4%	115 €
Thoracotomy	21.3%	4437 €
**Total cost (mean per person)**		2110 €

Abbreviations:, CT, computed tomography.

Supplementary Table S4. Cost-Utility Analysis of the Annual and Biennial LDTDT-Scan Screening Strategies.

Strategy	Age at onset	Smoking AND asbestos exposure		Smoking		High asbestos exposure		Asbestos exposure	
		Time horizon		Time horizon		Time horizon		Time horizon	
		10 yr	Lifetime	10 yr	Lifetime	10 yr	Lifetime	10 yr	Lifetime
**Annual LDTDT**									
	50 yr	419,247	103,039	417,438	117,769	670,873	152,324	747,661	170,485
	55 yr	274,345	90,624	394,322	114,854	584,079	146,952	720,257	171,575
	60 yr	182,968	90,809	411,857	117,955	531,025	155,982	803,737	187,957
**Biennial LDTDT**									
	50 yr	108,027	47,661	107,652	52,179	160,950	61,387	177,237	66,386
	55 yr	89,035	45,331	102,262	49,195	147,498	58,743	167,985	64,023
	60 yr	60,622	41,597	115,710	51,099	145,898	60,170	197,306	69,005
		**Pleural plaque(s)**		**Asbestosis**		**Smokers and former smokers**		**Intermediate asbestos exposure**	
		Time horizon		Time horizon		Time horizon		Time horizon	
		10 yr	Lifetime	10 yr	Lifetime	10 yr	Lifetime	10 yr	Lifetime
**Annual LDTDT**									
	50 yr	791,111	167,606	482,004	112,202	642,683	167,076	774,334	173,469
	55 yr	690,974	157,823	319,788	99,531	740,137	185,396	730,670	174,300
	60 yr	633,257	157,215	214,072	101,620	1,552,823	231,089	778,404	193,499
**Biennial LDTDT**									
	50 yr	186,464	65,916	121,105	50,067	154,979	65,146	182,901	67,196
	55 yr	170,732	60,790	100,235	48,011	155,809	65,146	172,070	65,241
	60 yr	169,697	61,333	69,183	44,366	267,433	79,123	194,727	70,090

Values are expressed in Euros.
LDTDT, low-dose thoracic computed tomography scan.
